# The Impact of Autoantibodies on IVF Treatment and Outcome: A Systematic Review

**DOI:** 10.3390/ijms20040892

**Published:** 2019-02-19

**Authors:** Mara Simopoulou, Konstantinos Sfakianoudis, Evangelos Maziotis, Sokratis Grigoriadis, Polina Giannelou, Anna Rapani, Petroula Tsioulou, Agni Pantou, Theodoros Kalampokas, Nikolaos Vlahos, Konstantinos Pantos, Michael Koutsilieris

**Affiliations:** 1 Department of Physiology, Medical School, National and Kapodistrian University of Athens, 75, Mikras Asias, 11527 Athens, Greece; vagmaziotis@gmail.com (E.M.); sokratis-grigoriadis@hotmail.com (S.G.); lina.giannelou@gmail.com (P.G.); rapanianna@gmail.com (A.R.); petroulatsi@yahoo.gr (P.T.); mkoutsil@med.uoa.gr (M.K.); 2 Assisted Conception Unit, 2nd Department of Obstetrics and Gynecology, Aretaieion Hospital, Medical School, National and Kapodistrian University of Athens, 76, Vasilisis Sofias Avenue, 11528 Athens, Greece; gynoffice04@gmail.com; 3 Centre for Human Reproduction, Genesis Athens Clinic, 14-16, Papanikoli, 15232 Athens, Greece; sfakianosc@yahoo.gr (K.S.); agnipantos@gmail.com (A.P.); info@pantos.gr (K.P.); 4 Aberdeen Maternity Hospital, Fertility Center-Assisted Reproduction Unit, Aberdeen AB25 2ZL, UK; t.kalampokas@abdn.ac.uk

**Keywords:** auto-antibodies, autoimmune disorders, assisted reproduction, in vitro fertilization

## Abstract

The role of autoantibodies in in vitro fertilization (IVF) has been discussed for almost three decades. Nonetheless, studies are still scarce and widely controversial. The aim of this study is to provide a comprehensive systematic review on the possible complications associated to autoantibodies (AA) impeding the chances of a successful IVF cycle. An Embase, PubMed/Medline and Cochrane Central Database search was performed on 1 December 2018, from 2006 until that date. From the 598 articles yielded in the search only 44 relevant articles ultimately fulfilled the inclusion criteria and were qualitatively analyzed. Five subsets of results were identified, namely, thyroid related AA, anti-phospholipid antibodies, anti-nuclear antibodies, AA affecting the reproductive system and AA related to celiac disease. It may be implied that the majority of auto-antibodies exert a statistically significant effect on miscarriage rates, whereas the effects on clinical pregnancy and live birth rates differ according to the type of auto-antibodies. While significant research is performed in the field, the quality of evidence provided is still low. The conduction of well-designed prospective cohort studies is an absolute necessity in order to define the impact of the different types of autoantibodies on IVF outcome.

## 1. Introduction

The evolution of assisted reproductive technologies (ART) from classic in vitro fertilization (IVF) [1] and intracytoplasmic sperm injection (ICSI) [2] to the era of prediction models employing artificial intelligence [3] over the past years has encouraged a worldwide reproductive revolution. The etiology of infertility is thought to be multifactorial with some of the key aspects being genetic abnormalities of equally male and female origin, ovulatory disorders, tubal obstructions, uterine, or peritoneal issues linked to female infertility and male factor associated with poor sperm quality [4]. In 2018 the European Society of Human Reproduction and Embryology (ESHRE) reported that 20–30% of infertility cases are attributed to physiological causes in men, 20–35% to female etiology, and 25–40% are related to both female and male factor infertility. The remaining 10–20% of infertility cases [5] are categorized as unexplained or idiopathic cases and most of these couples suffer from recurrent implantation failure (RIF). Despite ART’s remarkable development there is still a significant percentage of failed IVF attempts. Numerous existing reports have focused on various factors, from uterine anatomy and endometrium receptivity, to connective tissue disorders and several immunologic factors, impacting negatively or positively the success rates of IVF treatments [6,7].

The process of implantation represents a critical step involving the interaction between the embryo and uterine epithelium [8]. During implantation two immunologically and genetically distinct tissues are challenged into achieving successful communication. In the current bibliography, several autoimmune factors have been associated with implantation failure outcomes [8,9,10]. In order to investigate reproductive failure, certain studies focused on associations between the autoimmune system and the IVF/ICSI outcome highlighting the role of autoantibodies during treatment [8,11]. Furthermore, recently it has been suggested that autoimmune diseases, such as systematic lupus, erythematosus, and anti-phospholipid syndrome, play a crucial role in infertility and its management. This relationship is established either through a direct association between autoimmune disorders, compromising an otherwise good fertility status, or autoimmune disorders adding another level of complexity to an existing poor fertility status. 

Interestingly, the levels of antiphospholipid antibodies (APL), antinuclear antibodies (ANA), or thyroid auto-antibodies (TAA) appear to be significantly increased in women diagnosed with unexplained infertility. Additionally, serum auto-antibodies are associated with early ovarian failure thus their contribution to infertility remains a topic of a heated debate [10,11]. Furthermore, anti-sperm antibodies are more often associated with fertilization failure when found in high titers in seminal plasma [8]. The role of autoantibodies in IVF has been debated for almost three decades and still global literature lacks the clinical evidence in order to delineate their role in infertility and standardize respective management. With practitioners facing conundrums in managing infertility where autoimmune antibodies are involved, and the lack of universal protocols recruited to overcome complex cases in the spectrum of autoimmune disorders, it is imperative for the scientific community to pursue the search for the holy-grail in understanding and subsequently successfully treating these multifaceted cases.

The aim of this study is to extract evidence-based data from current published literature in order to provide information pertaining to the patients’ performance that may be anticipated following IVF treatment in relation to the presence of autoimmune antibodies. Herein a systematic review attempts to provide a comprehensive analysis on the possible associations of autoantibodies in regards to a successful IVF cycle. The analysis revolves around the most prevalent groups of autoantibodies that have been reported to affect the reproductive system including thyroid autoantibodies, antiphospholipid autoantibodies, antinuclear autoantibodies, antisperm, antiendometrial, antigonadotropin autoantibodies, and autoantibodies causing celiac disease. Respective associations in regards to IVF outcome and conclusions pertaining to subsequent management constitute the driver of this work.

## 2. Materials and Methods

### 2.1. Search Strategy

**Figure 1:**
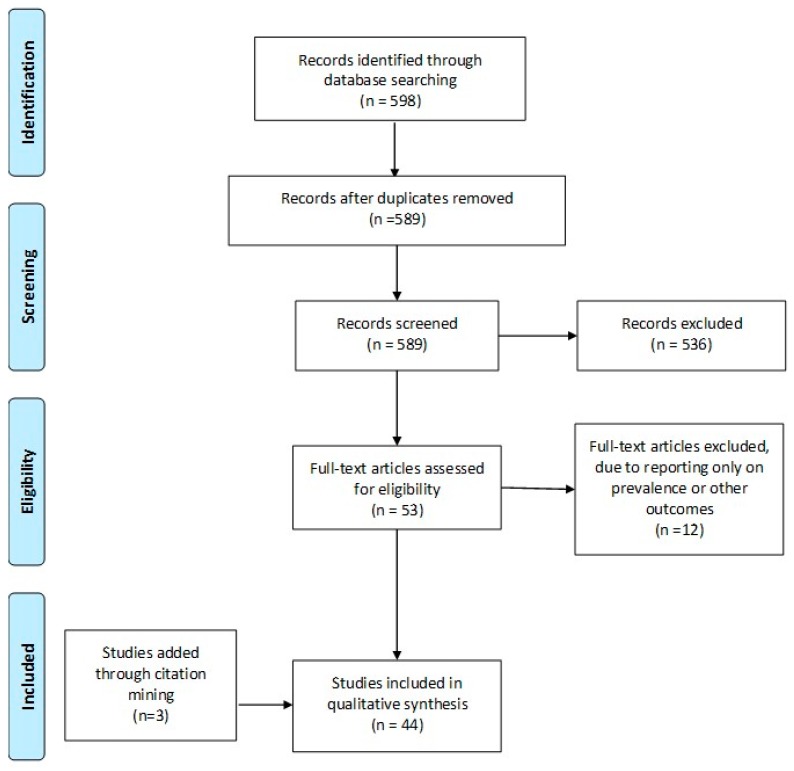
PRISMA flowchart.

A systematic search of the literature was performed in Pubmed/Medline, Embase, and Cochrane Central databases on the 1 December 2018, from 2006 until that date (Supplementary Materials). The keywords employed and combined for the search strategy were: “In-Vitro Fertilization”, “IVF”, “Assisted Reproduction”, “Assisted Reproduction Techniques”, “Medical Assisted Reproduction”, “Intracytoplasmic Sperm Injection”, “ICSI”, “auto-antibodies”, “antibodies”, “anti-sperm”, “antinuclear antibodies”, “anti-thyroid antibodies”, “autoimmune disorders”, “autoimmunity”. The original search yielded 598 studies from the three databases. Following the removal of duplicate studies (*n* = 9), all records were screened and full-text was sought and obtained for relevant articles. Relevant articles (*n* = 53), were identified following title and abstract screening, employing the flow chart of Preferred Reporting Items for Systematic Reviews and Meta-analysis (PRISMA) as presented in Figure 1. Screening and selection of literature was performed independently by three authors. Disagreements between the authors were resolved by an arbitration mediated by the senior authors. Citation mining was performed where the reference lists of all included articles and relevant reviews and metanalyses were reviewed to identify other articles of relevance. The search was limited to full-length manuscripts published in English in peer-reviewed journals up to December 2018. A total of 44 studies were included in the present systematic review. No protocol was submitted to the Prospero International Prospective Register of Systematic Reviews, providing details on conducting of this study.

### 2.2. Study Selection

Only studies that were performed following 2006 were included. As evidenced by the majority of literature, IVF from inception until 2006 reported continuous improvements regarding live birth rates [12]. Since 2006, live birth rates reached a plateau with adjustments reported each year. The population of the study included women undergoing IVF. The primary outcome measure was live birth rate and/or ongoing pregnancy (LB/OP). Both LB and OP were included, as many studies report on different findings and there is a lack of consensus on the desired outcome [13,14]. Secondary outcome measures were clinical pregnancy rate (CP), biochemical pregnancy rate (BP) and miscarriage rate. Miscarriage rate is calculated in regards to clinical pregnancy.

### 2.3. Data Extraction

Even though data extraction is not commonly entailed in a systematic review, the authors decided to perform data extraction as the definition for each outcome was presented with great variety among the studies, ranging significantly from live-birth per cycle to live-birth per clinical pregnancy. The reporting of outcomes in a single-widely accepted-definition is of pivotal importance for the study to be comprehended. The authors herein define the biochemical, clinical pregnancy and live birth/ongoing pregnancy rates as the number of the aforementioned per woman per cycle. The miscarriage rate is defined as the number of patients’ miscarriages per patients achieving a clinical pregnancy.

## 3. Thyroid Related Autoantibodies

It is well established in literature that thyroid dysfunction could jeopardize fecundity via several pathophysiological mechanisms. The hypothalamic-pituitary-thyroid (HPT) axis affects directly the function of the hypothalamic-pituitary-ovarian (HPO) axis, and vice versa. As a result, the two axes act together as an incorporated system. The physiological communication between HPT axis and HPO axis is mainly mediated mainly by a number of specific thyroid hormone receptors existing in the ovaries. Further to that, there is sufficient data demonstrating that estrogen directly affects the HPT axis functionality in the hypothalamus-pituitary level [15]. This incorporation is reflected in the fact that both hyperthyroid and hypothyroid women suffer from menstrual disturbances and anovulatory cycles, conditions that equally compromise fertility [16,17].

The main cause leading to thyroid dysfunction is thyroid related auto-immunity. It is demonstrated that in women presenting with thyroid autoimmunity, namely Grave’s disease and Hashimoto thyroiditis, the prevalence of infertility was very high and reached 47% and 52%, respectively [18]. In a cross-sectional study nested within an ongoing prospective cohort study, the prevalence of thyroid auto-immunity in a cohort of infertile women was investigated. The results demonstrated that the prevalence of thyroid auto-immunity was statistically significant higher in the infertile group (19%) in comparison to the control group consisting of fertile women (13%). Furthermore, women with thyroid auto-immunity presented with a statistically significant higher serum thyroid stimulating hormone (TSH) and thyroid globulin auto-antibodies (Tg-Abs) levels in comparison to women without thyroid auto-immunity [19]. Other studies suggest that infertile women present with higher chances to be positive to anti-thyroperoxydase antibodies (anti-TPO) in comparison to age-matched fertile controls, even if they are euthyroid [20]. Additionally, several other published data provide indications regarding the possible relationship between thyroid auto-immunity and infertility [21]. Nonetheless, there is la ack of robust data to provide evidence for a causal relationship between thyroid auto-immunity and infertility and several dilemmas arise for the practitioners regarding the management of this special group of infertile patients during assisted reproduction treatment (ART) [22]. There are insufficient data regarding the direct impact of thyroid auto-antibodies (TAA) on IVF outcome, especially for TAA-positive euthyroid women. 

Fourteen studies were considered suitable for inclusion in this systematic review as in all of them the possible effect of TAA in IVF outcome was investigated (Table 1) [9,11,23,24,25,26,27,28,29,30,31,32,33,34]. Data extraction was performed to provide information regarding the studies’ general characteristics, the characteristics of the studies’ groups and the IVF outcomes presented (Table 1). Eight out of 14 studies included in this review performed a retrospective data analysis [9,24,27,28,30,31,33,34] and the other six were prospective cohort studies [11,23,25,26,29,32].

Three studies report on BP rate [26,29,32]. No statistically significant difference was observed. Fourteen studies reported on CP rates [9,11,23,24,25,26,27,28,29,30,31,32,33,34]. Eight of the aforementioned studies were of retrospective nature and the remaining five of prospective. Only three studies observed a statistically significant lower CP rate in the TAA positive group [9,23,26]. These findings are in accordance with three previous meta-analyses performed in the field [35,36,37]. 

**Figure 2:**
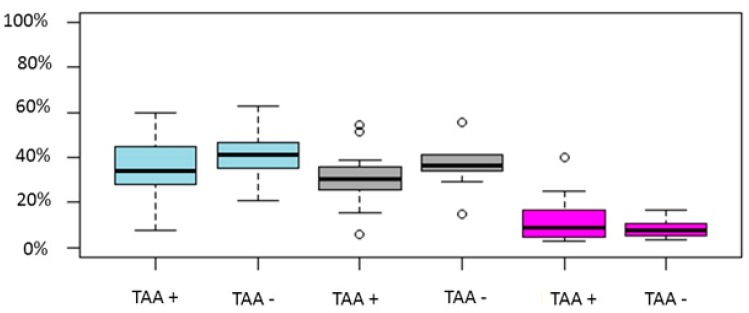
Boxplot regarding CP, LB/OP and miscarriage rates between TAA positive and TAA negative women. Blue color represent CP rate, grey LB/OP rate and magenta represent miscarriage rates. Circles in the figure represent outliers -values outside the 1.5x interquartile range.

Eleven studies reported on miscarriage rate [9,11,24,25,27,29,30,31,32,33,34]. Only one study reported a higher miscarriage rate in the TAA positive group, while the remaining ten demonstrated no statistically significant difference. The increase in miscarriage rate is also demonstrated in two of the three meta-analyses [35,37]. This is attributed to the sample size as evident in Busnelli’s meta-analysis. Ten studies reported results on LB/OP rate [9,11,24,25,27,30,31,32,33,34]. Seven of the included studies were of retrospective nature and the remaining three of prospective. The sample size ranged from 82–1239 cycles. No study reported a statistically significant difference in LB/OP rates. Only one out of three meta-analyses reported lower LB/OP rates. It may be possible that a well-designed prospective cohort study will delineate the possible impact of TAA on LB/OP in IVF cycles. The sourced data for each of the above outcomes is graphically presented in Figure 2.

In conclusion, following evaluation of the evidence published from 2006 to 2018, TAA presence in euthyroid women appears not be correlated with poorer outcomes following IVF/ICSI cycles. Regarding the miscarriage rates, the conclusions are conflicting and the consensus debatable, as there is data demonstrating that the miscarriage rate is not correlated with TAA presence, while other studies support the opposite, as buttressed by a meta-analysis published by Toulis et al. (2010) [35]. Nonetheless, the maternal TAA positivity could impair IVF/ICSI outcomes when overt thyroid dysfunction or subclinical hypothyroidism are co-existent. It should be highlighted, that studies included in this systematic review have enrolled populations of varying characteristics and diverse infertility etiologies. Thus, the level of heterogeneity among them is assessed as being considerably high. Further to that, the included studies herein, employed dissimilar cut-off levels and understandably entail different laboratory methods in order to evaluate the thyroid hormonal profile, this may serve as a consideration. Regarding infertility treatment, numerous IVF/ICSI treatment protocols have also been employed among the different studies. The main limitation observed in the great majority of the studies and especially in consideration to the prospective studies, is the small sample size of the studied populations. As a result, the strength of the provided evidence within the published literature is restricted. Robust data provided by future larger prospective studies and meta-analyses would provide more robust evidence in order to address the possible effects of the TAA in IVF/ICSI outcome. This will contribute considerably, towards a consensus and subsequent implementation of a common universal protocol on the optimal management of the euthyroid TAA positive women in assisted reproductive medicine. Data provided in this study are in the same line with these provided by others studies in the field [10,36] and concordant with the current Guidelines of the American Thyroid Association for the Diagnosis and Management of Thyroid Disease During Pregnancy and the Postpartum published in 2017 [22]. 

## 4. Anti-Phospholipid Antibodies

The Anti-Phospholipid Syndrome (APS), diagnosed by the presence of anti-phospholipid antibodies (aPL) has been associated with various pregnancy-related complications, such as pre-eclampsia, still birth, pre-term delivery and miscarriages [38,39,40]. Numerous studies have linked aPL to infertility, though safe conclusions cannot be reached yet [10]. In 2017 EULAR guidelines have been published regarding the management of patients with APS and/or Lupus [41]. According to guidelines ART is a safe procedure for the patient, although precautious treatment entailing low-dose aspirin, heparin, and antithrombotic treatment is suggested. The employment of natural cycles to avoid ovarian hyperstimulation syndrome is mentioned but due to the lower success rates no recommendation has been published. The stimulation protocols that should be employed are the ones suggested by Bellver and Pellicer [42].

Fifteen studies were identified including anti-Phospholipid antibodies and IVF outcome [11,43,44,45,46,47,48,49,50,51,52,53,54,55,56]. Most of the included studies were of prospective nature, while three of retrospective, one1 case report, and two case series were included. Data extraction was performed with respect to provide information regarding to the studies’ general characteristics, the characteristics of the studies’ groups and the IVF outcomes presented (Table 2).

Out of 12 cohort studies, 11 employed aPL-negative women as controls [11,43,44,47,48,51,52,53,54,55,56] and one employed women with Lupus as control group [46]. Regarding cohort studies employing aPL-negative women as controls, seven studies reported on LB/OP rate [11,43,44,47,53,54,55], ten studies reported on clinical pregnancy rate [11,43,44,47,51,52,53,54,55,56] and eight studies on miscarriage rate [11,43,44,47,51,52,53,54]. One study reported only on biochemical pregnancy (positive pregnancy test) [48]. 

Regarding the case report by Andreeva and colleagues [45], the authors presented the case of an infertile female patient negative for APS by standard diagnostic protocol, but diagnosed with Systemic Lupus Erythematosus (SLE). Following further evaluation, the patient was positive for IgA Anti-β2glycoprotein I Antibodies, suggesting APS. The patient achieved a pregnancy following the first IVF cycle, indicating that an IVF cycle may present an approach for APS and SLE patients. The case-series by Ulcova-Gallova [50] suggests the employment of PGD for patients with APS and repeated pregnancy loses. Finally, the case series by Ragab and colleagues suggested the employment of ART for infertile patients with APS. Out of five patients enrolled in the study one achieved a pregnancy thus opening a new line of approach for these patients [49]. 

The study by Orquevaux and colleagues [46] investigated the safety of IVF when performed on patients with SLE and/or APS. Thirty-seven patients underwent a total of 97 cycles. Twenty-six out of 37 achieved a healthy delivery, whereas only 8 cycles resulted in complications. Similarly, Da Costa’s study evaluated the effects of two aPLs, namely anticardiolipin antibody and lupus anticoagulant in women undergoing IVF. Comparable biochemical pregnancy rates were observed regarding aPL-positive (*n* = 30) and aPL-negative women (*n* = 181). The study did not report on clinical, ongoing pregnancy, or live birth rates. 

**Figure 3:**
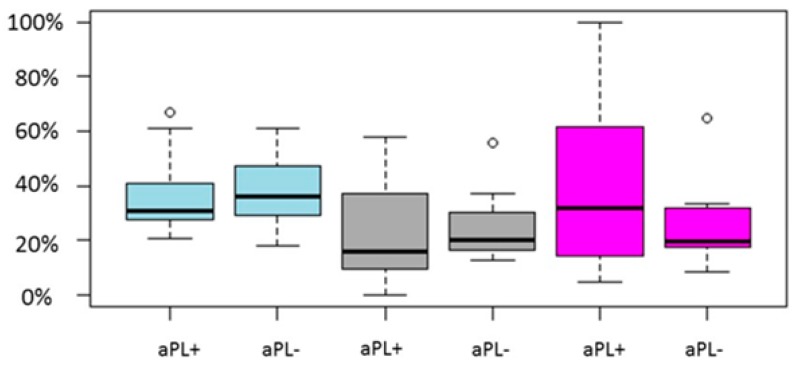
Boxplot regarding CP, LB/OP and miscarriage rates between aPL positive and aPL negative women. Blue color represent CP rate, grey LB/OP rate and magenta represent miscarriage rate. Circles in the figure represent outliers -values outside the 1.5x interquartile range.

Regarding the 10 studies that presented with aPL-negative women serving as the control group, the study size ranged from 40–1239 cycles. The sample size of cycles with aPL-positive women ranged from 8 to 395, whereas the control group size ranged from 27–844 cycles. No study presented with statistically significant different LB/OP rate. Only two studies presented with a statistically significant lower clinical pregnancy rate for aPL-positive women [51,52]. Both studies were of retrospective nature. The pooled results of the studies are graphically presented in Figure 3.

In conclusion, as reported by the studies analyzed herein, the presence of aPL does not appear to influence neither ongoing pregnancy/live birth rates nor clinical pregnancy rates. The consideration that pooled results may alter the outcome should not be overlooked. The option of a meta-analysis including the current studies was rejected as it would lead to low quality evidence, thus constituting it more confusing than delineating. As anticipated, a higher miscarriage rate was observed in the majority of the reports. The fact that the presence of aPL did not decrease pregnancy rates is of pivotal importance as it indicates both the safety and the efficacy of IVF for infertile women diagnosed as aPL-positive. Nonetheless, it should be highlighted that IVF cycles that corresponded to women positive for aPL represented solely one quarter in comparison to the control. This fact could be attributed to the estimated prevalence of aPL in the population of women diagnosed with unexplained or immunological-based infertility.

## 5. Antinuclear Antibodies-ANAs

Antinuclear antibodies (ANAs) are auto-antibodies related to systematic autoimmune disorders. They constitute a large group of auto-antibodies targeting several cellular antigens such as double-strand DNA (ds-DNA), RNA molecules, mitochondria antigens, several proteins in the cytoplasm and in the nucleus, and their complexes. Among the types of ANAs are the anti-ds DNA antibodies, anti-centromere antibodies (ACA) and the anti-extractable nuclear antigens antibodies (anti-ENAs) [58,59]. It is well documented that ANAs are implicated to the pathogenesis of several systematic autoimmune disorders [60,61,62,63,64,65,66,67]. Evidence provided from the literature indicates that the presence of ANAs is also associated with immunologically induced infertility. However, the underlying mechanisms leading to this association are to date under investigation. There is data indicating that women with high levels of ANA in their sera have also elevated ANA levels in their follicular fluids and these levels are documented to be negatively correlated with the number of good quality embryos obtained in IVF/ICSI cycles [68]. Further to that, in vivo experiments employed in mouse models demonstrate that the presence of ANAs in the oocytes and in the embryos’ culture media could compromise oocyte maturation and embryo development, respectively [68,69]. These observations, lead the researchers to conclude that ANAs could directly compromise oocyte maturation and embryo development leading finally to infertility [70,71,72].

In several studies the prevalence of ANA is recorded to be higher in women presenting with fertility disorders than in fertile women. Interestingly, as indicated from a large cohort study, the presence of ANA is negatively correlated with parity [73,74,75]. Nonetheless, there are still insufficient data regarding the direct impact of ANAs on IVF/ICSI outcome, especially for ANA positive women presenting with an undisturbed immunologic profile.

Seven studies were considered suitable for inclusion in this systematic review as in all of them the possible effect of ANAs in IVF/ICSI outcome was investigated [11,68,76,77,78,79,80]. Data extraction was performed to provide information regarding the studies’ general characteristics, such as year of publication, type of the study, type of ANAs investigated, along with the characteristics of the studies’ groups and the IVF/ICSI outcomes presented, referring to biochemical pregnancy rates, clinical pregnancy rate, live birth rate, miscarriage rate (Table 3). Four out of seven studies included in this review performed a retrospective data analysis and the other three were prospective cohort studies. However, it should be mentioned that the authors in four out of the seven studies [68,76,77,78] did not provide exact information regarding the nature of the studies-whether prospective or retrospective- and thus conclusions were drawn from information provided in regards to the patients’ consent statements.

Five out of seven studies included in this systematic review investigated the impact of ANAs in the IVF/ICSI outcome, considered ANAs as a single category of autoantibodies. Two out of five studies were of retrospective nature [68,80], while the remaining three were prospective [11,77,78]. In all of these studies, the study group consisted of sero-positive for ANA (ANA+) infertile women while sero-negative for ANA (ANA−) women were serving as a control. All women participated were infertile women presenting with different types of infertility etiologies. Etiologies ranged greatly from women suffering from autoimmune disorders to women who had a history of a known medical treatment or surgeries that could compromise fertility were excluded. The studies’ size ranged from 100–1022 cycles. The sample size of cycles with ANA positive women ranged from 50 to 202, whereas the control group size ranged from 50–844 cycles. Regarding the IVF/ICSI outcomes investigated in this systematic review, three studies reported on live birth rate/ongoing pregnancy rates, five studies reported on clinical pregnancy rates, and four studies on miscarriage rate. Only two studies reported on positive hCG test rates.

**Figure 4:**
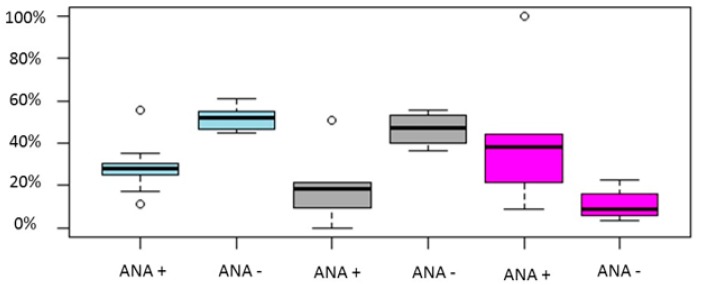
Boxplot regarding CP, LB/OP and miscarriage rates between ANA positive and ANA negative women. Blue color represents CP rate, grey LB/OP rate and magenta represents miscarriage rates. Circles in the figure represent outliers -values outside the 1.5x interquartile range.

Regarding live birth/ongoing pregnancy rates a noteworthy difference was observed between ANA positive group and ANA negative group in two out of three studies [77,80], while, in the study of Chen et al. (2017) [11] no statistically significant difference could be established between the two groups. ANA positive women presented with statistically significant decreased clinical pregnancy rate in four out five studies [68,77,78,80]. Similar to live birth/ongoing pregnancy rates, in the study of Chen et al. (2017) [11] clinical pregnancy rate did not differ significantly between the two groups. In both studies presenting data regarding biochemical pregnancy rate, the ANA positive group presented with a statistically significant decreased positive hCG test rate compared to ANA negative group [77,78]. In regards to miscarriage rates, the results are controversial. According to Li et al. (2015) and Zhu et al. (2013) [77,80] ANA positivity was correlated with higher miscarriage rates, while in the studies of Chen et al. (2017) and Ying et al. (2012) such a correlation could not be established [11,68]. Data regarding the live birth rate/ongoing pregnancy rate, the clinical pregnancy rate, the positive hCG test rate and the miscarriage rate is provided in Table 3. Pooled results of the above outcomes are graphically represented in Figure 4.

Further to that, some studies indicate that ANAs presence could impair the fertilization rate and the number of good quality embryos and thus could lead to IVF/ICSI failure [68,78,80]. In addition, there are indications that the presence of specific ANAs subclasses, namely Anti-Rib-p, anti-Jo-1, and anti-dsDNA antibodies, correlate with a high risk of implantation failure and early miscarriages in women undergoing IVF/ICSI treatment [77]. The same study documented an observation of high significance, namely that not only infertile women but similarly fertile women could be ΑΝΑ positive. Nevertheless, only infertile women presented with a high ANA titer (>1:320) [77]. In the study of Zhu et al. (2013), the effectiveness of the prednisolone plus low-dose aspirin adjuvant treatment for ANA positive women on IVF/ICSI outcome was investigated. Study results indicate that ANA positive women who were treated with adjuvant treatment employing prednisolone plus low-dose aspirin, exhibited a significantly better overall IVF performance in comparison to the untreated patients. Authors highlighted that the prednisolone plus low-dose aspirin administration could be beneficial for ANA positive women undergoing IVF treatment. Due to the retrospective nature and the small size of participants included in some of the groups, this study involved a high risk of bias. Consequently, the study’s outcomes should be verified by large, randomized-controlled trials prior to application in clinical practice [80].

The remaining two out of the seven studies included in this systematic review investigated the impact of specific ANAs subcategories in the IVF/ICSI outcome [76,79]. Data regarding the type of ANAs investigated, the live birth rate/ongoing pregnancy rate, the clinical pregnancy rate, the positive hCG test rate and the miscarriage rate are provided in Table 3. 

In the first study, Fan et al. (2017) [76] retrospectively investigated the impact of anti-dsDNA autoantibodies on IVF outcome. Fifty-two women with positive ANA and anti-ds DNA antibodies (ANA positive/anti-ds DNA positive group) were compared to 86 women with positive ANA and negative anti-ds DNA antibodies (ANA positive/anti-ds DNA negative group) and 121 women with negative ANA and anti-ds DNA antibodies (ANA negative/anti-ds DNA negative group). Patients positive for other autoantibodies, such as thyroid autoantibodies or anticardiolipin antibodies, or patients with autoimmune diseases or clinical presentations of autoimmune diseases were excluded from the study. ANA positive/anti-ds DNA positive group was presented with statistically significant decreased clinical pregnancy rate and statistically significant increased miscarriage rate compared with both ANA positive/anti-ds DNA negative group and ANA negative/anti-ds DNA negative group. In addition, data extraction revealed that the live birth rate/ongoing pregnancy rate was also noteworthy decreased in the ANA positive/anti-ds DNA positive group compared with both ANA positive/anti-ds DNA negative group and ANA negative/anti-ds DNA negative group, as no live birth/ongoing pregnancy could be achieved in ANA positive/anti-ds DNA positive. Authors concluded that ANA presence may be an essential marker for oocyte quality and embryo development in infertile women positive for any type of ANA. It should be highlighted that authors do not provide clear evidence regarding the distribution of infertility etiologies among the study’s groups, a parameter which possess the major limitation of this study.

In the second study published from the same team as the first one, Ying et al. (2013) [79] retrospectively investigated the impact of ACA on oocyte maturation and embryo development in ICSI cycles. Twenty women with positive ANA and ACA (ANA positive/ACA positive group) were compared to 51 women with positive ANA and negative ACA (ANA positive/ACA negative group) and 116 women with negative ANA and ACA (ANA negative/ACA negative group). Women who presented with a history of a known medical treatment or surgeries that could compromise fertility were excluded from the study. ANA positive/ACA positive group presented with a statistically significant decreased number of high-quality embryos, biochemical and clinical pregnancy rates compared with ANA negative/ACA negative group. Authors concluded that ACA presence may be an essential marker for oocyte quality and embryo development in infertile women positive for any type of ANA undergoing ICSI treatment. It should be noted that in the present study the possible co-existence of other autoantibodies was not considered an exclusion criterion-unlike the study of Fan et al. (2017) [76] and, hence, it should be considered as a serious confounding factor.

In conclusion, ANAs presence is probably correlated with poor outcomes following IVF/ICSI cycles. The great majority of the studies are in line with the conclusion that ANAs positivity is correlated with lower clinical pregnancy rates and higher miscarriage rates. Further to that, studies indicate that ANAs could adversely affect oocyte quality and embryo development leading to infertility and possibly to immunologically induced RIF [68,81]. However, it should be mentioned that ANAs positivity was not only reported on infertile women population but also in fertile populations. Due to the fact that ANAs constitute a large group of different autoantibodies, future studies evaluating the effect of specific ANA types are required in order to provide guidance to the clinicians. There are insufficient data regarding the efficacy of adjuvant treatments for ANA positive women on IVF/ICSI outcome [10]. It should be highlighted, that studies included in this systematic review have enrolled populations of varying characteristics and diverse infertility etiologies. Thus, the level of heterogeneity among them is assessed as being considerably high. Further to that, the included studies herein, employed dissimilar cut-off levels and understandably employed varying laboratory methods in order to evaluate the autoantibodies profile. As a result, the strength of the provided evidence within the published literature is restricted. Robust data provided by future larger prospective studies and meta-analyses would provide more robust evidence in order to address the possible effects of the ANA in IVF/ICSI outcome. The future studies should include patients only with abnormal levels of specific ANAs excluding any other condition, while the control group should be infertile patients of good prognosis, while the primary outcome measure should be the live birth rate.

## 6. Auto-Antibodies Specifically Affecting the Reproductive System

There is a wide range of auto-antibodies that affect the reproductive system. A common cause of male infertility is the anti-sperm antibodies (ASA) that are present in the semen. ASA may be present in the follicular fluid but are not related to auto-immunity, thus female ASA were excluded from the present study. Auto-antibodies affecting the female reproductive system are anti-gonadotropin antibodies (AGA), anti-endometrial antibodies (AEA) and anti-laminin-1 (aLN1) antibodies present in the follicular fluid.

Nine studies were identified affecting the reproductive system and possibly altering the possibilities for a successful IVF cycle [82,83,84,85,86,87,88,89,90]. Four of the aforementioned studies concerned ASA [87,89,90,91], 3 AEA [83,86,88], one AGA [82] and one aLN1 in the follicular fluid of women with endometriosis [85]. Although ALN1 is not classified as a reproductive-specific antibody, the study population is diagnosed with endometriosis, a conditioned that has been associated to aLN1 [92]. Characteristics of the studies are presented in Table 4.

Two out of four studies regarding ASA were of retrospective nature, while the remaining two were prospective. Only two studies provided LB/OP data. The control group in one of those studies were oligoasthenoteratozoospermic (OAT) men. According to the study men with ASA had a better ICSI cycle outcome compared to OAT men [87]. Two studies reported results in clinical pregnancy [90,91]. The control groups were men without ASA. No statistically significant difference between the two groups was observed in either of the studies. A meta-analysis was performed on ASA [84] and the authors concluded that seminal ASA do not impede pregnancy possibilities. A limitation of that meta-analysis was the different cut-off points employed by the studies examined (ranging from 1% to 80%).

Regarding AEA, three studies were identified, all of them being of prospective nature. No study reported on LB/OP data. Two studies reported on clinical pregnancy. The study by Saparik and colleagues [86] investigated a possible correlation between the molecular weight of IgA, IgG, and pregnancy outcome. According to the results of that study, IVF outcome may be negatively correlated with certain subtypes of AEA. Examining another perspective, the presence of AEA in the sera of female patients may impede their fecundity and the success of an IUI cycle [83]. In all three groups (with endometriosis, without endometriosis, and without laparoscopic investigation) of the study, women diagnosed as AEA positive were associated with a lower CP rate. A second IUI cycle though increased the chances of CP up to five times. Only one study reported on miscarriage rate. According to Randall and colleagues [88] AEA positive was correlated with higher miscarriage rates.

A case report was presented regarding AGA [82]. The patient was diagnosed with resistant ovary syndrome and AGA. Despite the unfavorable prognosis of the male partner (OAT) the patient achieved a pregnancy that lead to a live birth. This case report validates the approach of ART for infertile patients with AGA. The impact of aLN1 in follicular fluids was explored in a prospective cohort study [85]. Women positive for aLN1 presented with similar ongoing pregnancy rate as women diagnosed as aLN1-negative [85]. This is in contrast to the other study performed by the same team regarding aLN1 in serum of women with Hashimoto Thyroiditis [23].

In conclusion, most auto-antibodies affecting the reproductive system do not exert a negative correlation to IVF cycles outcome. An exception to this, are the AEA that are associated with lower clinical pregnancy rates and higher miscarriage rates. It should be highlighted that the studies in the field present with great heterogeneity. Most of them employ different assisted reproduction techniques, as evident by the employment of IVF, ICSI, or IUI for patients with similar infertility factors. The control group employed in some studies are also poor prognosis IVF patients posing as another limitation. Furthermore, the study group is commonly affected by an additional disorder other than the autoimmune, serving as a confounder regarding the potential towards achieving a pregnancy. The conduction of better designed observational studies is a necessity for the advancement of this field. The future studies should include patients only with abnormal levels of auto-antibodies excluding any other condition, while the control group should be infertile patients of good prognosis, and the outcome measure should be live birth. Following the conduction of an adequate number of observational studies for each of the above-mentioned auto-antibodies, a possible meta-analysis may delineate their role in infertility and the outcome of an IVF cycle.

## 7. Auto-Antibodies Related to Celiac Disease

Celiac disease (CD) is a chronic inflammatory autoimmune disorder originating by an abnormal adaptive immune reaction against gluten-containing grains in susceptible people [93]. The pathophysiological mechanisms leading to the disease include several genetic and environmental factors, and thus CD is considered to be a multifactorial disease, Regarding the pathophysiological mechanism leading to the disease, the great majority of the studies demonstrate that in the 90% of the patients present specific genes encoding for major histocompatibility (MHC) class II proteins including the human leukocyte antigen (HLA)-DQ 2 and HLA-DQ8. Both HLA DQ 2 and HLA-DQ8 are co-expressed on antigen-presenting cells (APCs). 

It is well documented that CD, except from the classic gastrointestinal symptoms characterized the disorder, is also correlated with several pathological conditions such as type-1 diabetes, autoimmune thyroiditis, autoimmune hepatitis and other forms of liver involvement, neurological disorders and dermatitis herpetiformis [93]. In addition, recently published studies correlate CD with reproductive disorders and infertility in both males and females [94]. Regarding females, it is considered that CD affects the female reproductive system via several pathological mechanisms leading to delayed menarche, amenorrhea, earlier menopause, recurrent abortions, recurrent implantation failures, hypogonadism, and negative pregnancy outcomes [95]. In a recent meta-analysis published by Tersigni et al. (2014) [96] interestingly women suffering from unexplained infertility, recurrent miscarriage or intrauterine growth restriction (IUGR) were presented with a statistically significant higher risk of CD when compared to the general population. The higher prevalence of CD among the women experiencing unexplained infertility was also confirmed by other studies [97]. Further to that, in a recent meta-analysis published by Singh et al. (2016) [98], infertile women presented with a statistically significant higher risk of having CD in comparison to the general population Nonetheless, there are insufficient data regarding the direct impact of auto-antibodies related to CD on IVF/ICSI outcome.

Only one study, published by Juneau et al. (2018) [99], was considered suitable for inclusion in this systematic review, as only in this study the possible effect of auto-antibodies related to CD with regards to IVF outcome was investigated. In this prospective cohort study, 28 sero-positive for CD women were compared to 967 sero-negative for CD women. Celiac disease positivity was defined as elevated (> 20 U/mL) levels of tissue transglutaminase IgA (tTG) or/and endomysial IgA (EMA) antibodies. Out of the 28 sero-positive women, 18 women submitted to a single-blastocyst stage embryo transfer and out of 967 sero-negative women, 724 underwent an embryo transfer. No statistically significant difference could be established between the two groups regarding the number of normal fertilized zygotes, the blastulation rate, the positive pregnancy hCG test rate (83.3% vs. 84.5%), the clinical pregnancy rate (50% vs. 69%), the live birth rate (50% vs. 68.1%) and the clinical pregnancy loss (27.8% vs. 17.5%). Additionally, the patients’ age, the hormonal profile (AMH levels), the number of oocytes retrieved and the number of transferred embryos did not differ significantly between the two groups. In the same study, authors employed a survey based on self-reports in order to investigate the possible effects of a gluten-free diet on IVF outcome, with gluten-free diet being to date the gold standard treatment for CD. No statistically significant difference was observed between the two groups regarding the patients’ dietary habits on IVF outcome. However, it should be highlighted that a significant number of the survey’s participants stated that they had experienced a previous miscarriage (38.8%) and suffer from menstrual irregularities (28%). In addition, the great majority of the participants stated that their diet contained gluten (91.4%). Authors, concluded that auto-antibodies related to CD did not impair IVF outcomes and the gluten-free diet did not present to have improved these outcomes. Despite the fact that this study in general could be characterized as well-designed, a number of serious limitations are identified. First of all, the sample size of the participants in the sero-positive for CD group was limited compared to the number of patients in the sero-negative group. A similar limited number of participants can be observed also in the group of patients stating that their diet was gluten-free (84 out of 987). In addition, authors employed per-protocol analysis in the sero-negative group, in contrast to the intention-to-treat analysis employed in the sero-positive group, regarding live birth rate. Furthermore, authors did not provide clear evidence regarding the distribution of the infertility etiologies among the study’s groups. It should also be noted that in the present study the possible co-existence of other autoantibodies was not an exclusion criterion. For these reasons, conclusions regarding the direct impact of the auto-antibodies related to CD on IVF outcome in cannot be safely drawn with respect to the data provided from this study.

In conclusion, there is insufficient data regarding the impact of auto-antibodies related to CD on IVF/ICSI outcome, as only one study investigated this relationship to date. Data provided in this study indicates that, probably, the screening for celiac disease may not be viewed as requirement for infertile women in general. However, several other studies demonstrate a higher prevalence of CD in women suffering from menstrual irregularities, unexplained infertility, recurrent miscarriages, recurrent implantation failures and IUGR compared to the prevalence of CD in the general population. These data indicate that auto-antibodies related to CD could adversely affect trophoblast invasion, finally leading to a poor placenta formation. Thus, a serologic screening for anti-endomysial and anti-TG antibodies may be suggested in such cases prior to IVF/ICSI treatment [97]. Robust data provided by future larger prospective studies and subsequent meta-analyses would provide more evidence in order to address the possible effects of the auto-antibodies related to CD on IVF/ICSI outcome. Future studies should include patients only with abnormal levels of anti-endomysial and anti-TG antibodies excluding any other condition, while the control group should consist of infertile patients of good prognosis, while the primary outcome measure should be the live birth rate.

## 8. Conclusions

In summary, while the effects of autoantibodies have been researched for many decades their effect on IVF cycle outcomes cannot yet be fully delineated. According to the literature search performed for this systematic review aPLs, TAA, and ASA do not seem to exert a negative outcome on IVF cycles regarding LB/OP, CP, and BP rate. On the other hand, AEA and ANA present with lower CP rates, hence inducing a negative effect. This, is certainly heightening the need for further ART cycles and, hence, the possibility of IVF overuse should be investigated.

It should be highlighted that most autoantibodies discussed, namely aPL, TAA, and ANA are associated with higher miscarriage rates. It may seem as a paradox that even though miscarriage rates are higher, there is no difference in live birth rates. This may be attributed to pure statistics. A possible explanation may be attempted in suggesting that the statistical significance is not reached due to sample size, while a possible pooling of the results may alter the outcome regarding LB/OP rates. The value that a meta-analysis may convey to the issue of delineating the role of autoantibodies in the spectrum of providing ART services would be considerable and, hence, it was investigated. The authors dismissed the idea of performing a meta-analysis as a number of the studies included herein have enrolled populations of varying characteristics and diverse infertility etiologies. Moreover, discrepant infertility treatment protocols have been employed thus increasing the covariates that must be accounted for. Another possible drawback is the different size both between the studies, as well as between the study and the control group employed within a study. Moreover, outcomes have been assessed differently by the numerous analyses employing varying approaches, adding to further confusion of the field. All of the above coupled by the fact that certain data originated form retrospective studies contributed to rendering a possible meta-analysis misleading.

To further our understanding and buttress current knowledge, conduction of well-designed prospective cohort studies is an absolute necessity in order to define the role of autoantibodies in the possibilities of IVF cycle success. A universal IVF treatment protocol, abiding to the proposed guidelines, as well as comparable laboratory techniques to evaluate the levels of autoantibodies should be employed. Blinding of personnel should also be considered by future research groups in order to enhance the robustness of the studies. Furthermore, a clear definition of the IVF outcome should be employed. Results should include only one cycle per patient and IVF outcome should be considered as clinical pregnancy and live birth rate or ongoing pregnancy—in case live birth reporting is not possible-per patient. The miscarriage rate should be reported per clinical pregnancy. Following the completion of such well-designed studies, future meta-analyses should be conducted in order to reach a final verdict regarding the role of autoantibodies in IVF cycle outcomes.

## Figures and Tables

**Table 1:** Study characteristics regarding thyroid auto-antibodies.

Studies	Type of Study	Type of Auto-Antibodies	Study Group	Control Group	Clinical Pregnancy Rate		Live Birth/Ongoing Pregnancy Rate		Miscarriage Rate	
					Study Group	Control Group	Study Group	Control Group	Study Group	Control Group
Chen et al., 2017 ^a^ [11]	Prospective	TPO-Ab, TG-Ab	235 TG-AB (+), 214 TPO-Ab (+)	844 TAA (−) women	59.6%, 54.2%	61%	54.4%, 51.4%	55.90%	5.1%, 2.8%	5.10%
Caccavo et al., 2016 [23]	Prospective	TPO-Ab, TG-Ab, Anti-laminin 1	44 Infertile Women affected by HT	28 Infertile Women without HT	9.1%	31.1%	n/a	n/a	n/a	n/a
Sakar et al., 2016 [32]	Prospective	TPO-Ab, TG-Ab	31 (32 cycles) TAA (+) women	121 (126 cycles) TAA (−) women	21.90%	20.60%	15.60%	15.10%	6.30%	5.50%
Lukaszuk et al., 2015 [27]	Retrospective	TPO-Ab	114 TAA (+) women	495 TAA (−) women	43.80%	47.50%	30.40%	34.10%	13.10%	13.30%
Tan et al., 2014 [33]	Retrospective	TPO-Ab, TG-Ab	110 TAA (+) women	725 TAA (−) women	40.9%	41.51%	39.09%	37.38%	4.44%	9.96%
Mintzori et al., 2014 [34]	Retrospective	TPO-Ab, TG-Ab	15 TAA (+) women	67 TAA (−) women	33.33%	37.31%	26.67%	34.32%	20%	8%
Chai et al., 2014 [24]	Retrospective	TPO-Ab, TG-Ab	89 TAA (+) women	419 TAA (−) women	44.90%	45.80%	32.60%	36%	9%	8.80%
Karacan et al., 2013 [25]	Prospective	TPO-Ab, TG-Ab	34 TAA (+) women	219 TAA (−) women	35.30%	40.60%	32.40%	37%	2.90%	3.60%
Magri et al., 2013 [28]	Retrospective	TPO-Ab, TG-Ab	60 TAA (+) women	202 TAA (−) women	28%	35%	n/a	n/a	n/a	n/a
Zhong et al., 2012 [9]	Retrospective	TPO-Ab, TG-Ab	90 TAA (+) women	676 TAA (−) women	33.3%	46.7%	24.33%	41.20%	8.97%	5.50%
Monteleone et al., 2011 [29]	Prospective	TPO-Ab, TG-Ab	14 TAA (+) women	17 TAA (−) women	n/a	n/a	n/a	n/a	40%	17%
Revelli et al., 2009 [31]	Retrospective	TPO-Ab, TG-Ab	52 TAA (+) women	200 TAA (−) women	7.6%	33%	6%	29%	25%	12%
Kilic et al., 2008 [26]	Cross-sectional and Prospective	TPO-Ab, TG-Ab	23 TAA (+) women	31 TAA (−) women	30.40%	41.90%	n/a	n/a	n/a	n/a
Negro et al., 2007 [30]	Retrospective	TPO-Ab	42 TAA (+) women	374 TAA (−) women	50%	62.60%	28.57%	55.34%	11.90%	7.20%

^a^: Chen et al., 2017, presents patients positive for more than one auto-antibody, thus cumulative results cannot be provided.

**Table 2:** Study characteristics regarding anti-phospholipid autoantibodies.

Studies	Type of Study	Type of Auto-Antibodies	Study Group	Control Group	Clinical Pregnancy Rate		Live Birth/Ongoing Pregnancy Rate		Miscarriage Rate	
					Study Group	Control Group	Study Group	Control Group	Study Group	Control Group
Di Nisio et al., 2018 [43]	Prospective	Lupus anticoagulant, Anti-cardiolipin antibodies, Anti- β2-glycoprotein antibodies	57 aPL (+) women	598 aPL (−) women	31.58%	29.26%	12.28%	20.23%	61.11%	30.86%
Hong et al., 2018 [44]	Prospective	Lupus anticoagulant, Anti-cardiolipin antibodies, anti-β2-glycoprotein 1 antibody	12 aPL (+) women	181 aPL (−) women	66.67%	45.86%	58.33%	37.02%	12.50%	19.28%
Andreeva et al., 2017 [45]	Case Report	IgA-anti-β2GPI	n/a	n/a	n/a	n/a	n/a	n/a	n/a	n/a
Chen et al., 2017 ^a^ [11]	Prospective	aCL-IgG, aCL-IgM, aβ2GPI-IgG, aβ2GPI-IgM	193 aCL-IgG (+) women, 202 aCL-IgM (+) women, 22 aβ2GPI-IgG (+) women, 246 aβ2GPI-IgM (+) women	844 aPL (−) women	58.0% 63.4% 36.4% 55.7%	61.02%	48.18% 53.96% 31.81% 50.81%	55.92%	17.00% 14.8% 12.50% 8.80%	8.35%
Orquevaux et al., 2017 ^b^ [46]	Retrospective	Lupus anticoagulant, Anti-cardiolipin antibodies, anti-β2-glycoprotein 1 antibody	12 SLE + aPL(+)/APS women 10 APS women	15 SLE + aPL (−) women	25%	32.43%	n/a	n/a	n/a	n/a
Paulmyer-Lacroix et al., 2014 [47]	Retrospective	Lupus anticoagulant, Anti-cardiolipin antibodies, IgA-anti-β2GPI	8 aPL (+) women	32 aPL (−) women	50.00%	53.13%	0.00%	18.75%	100.00%	64.71%
Da Costa et al., 2012 [48]	Retrospective	Lupus anticoagulant, Anti-cardiolipin antibodies	34 aPL (+) women	205 aPL (−) women	n/a	n/a	n/a	n/a	n/a	n/a
Ragab et al.,2012 [49]	Case Series	Lupus anticoagulant, Anti-cardiolipin antibodies	n/a	n/a	n/a	n/a	n/a	n/a	n/a	n/a
Ulcova-Gallova, 2012 [50]	Case Series	Anti-cardiolipin antibodies	n/a	n/a	n/a	n/a	n/a	n/a	n/a	n/a
Ying et al., 2012 [51]	Retrospective	Anti-cardiolipin antibodies	60 ACA (+) women	518 ACA (−) women	26.67%	44.98%	n/a	n/a	31.25%	15.88%
Zhong et al., 2011 [52]	Retrospective	Anti-cardiolipin antibodies	80 ACA (+) women	788 ACA (−) women	31.25%	48.60%	n/a	n/a	32.00%	19.32%
Lee et al., 2007 [53]	Prospective	Anti-cardiolipin antibody, lupus anticoagulant	39 aPL (+) women	142 aPL (−) women	20.51%	17.61%	7.69%	14.08%	62.50%	20.00%
Sanmarco et al., 2007 [54]	Prospective	Lupus anticoagulant, Anti-cardiolipin antibodies, anti-β2-glycoprotein 1 antibody	40 aPL (+) women	61 aPL (−) women	27.50%	19.67%	22.50%	13.11%	18.18%	33.33%
Buckingham et al., 2006 [55]	Prospective	Anti-cardiolipin antibodies, anti-β2-glycoprotein 1 antibody, phosphatidylserine	19 aPL (+) women	80 aPL (−) women	31.58%	36.25%	15.79%	23.75%	n/a	n/a
Matsubayashi et al., 2006 ^b^ [56]	Prospective	Anti-cardiolipin antibodies, anti-β2-glycoprotein 1 antibody,	17 aPL (+) women	27 aPL (−) women	29.41%	33.33%	n/a	n/a	n/a	n/a

^a^: Chen et al., 2017, presents patients positive for more than one auto-antiboy, thus cumulative results cannot be provided; ^b^: The data regarding the Matsubayashi et al., 2006 study were obtained from a letter to the editor by Matsubayashi and colleagues ([57]) for Buckingham et al., 2006 study.

**Table 3:** Study characteristics regarding Anti-nuclear auto-antibodies.

Studies	Type of Study	Type of Auto-Antibodies	Study Group	Control Group	Clinical Pregnancy Rate		Live Birth/Ongoing Pregnancy Rate		Miscarriage Rate	
					Study Group	Control Group	Study Group	Control Group	Study Group	Control Group
Chen et al., 2017 [11]	Prospective	−ANA	−178 ANA(+) Women	−844 ANA(-) Women	55.60%	61%	50.56%	55.90%	9.10%	8.30%
Fan et al., 2017 [76]	Not provided	−ANA −Anti-dsDNA	−52 ANA(+) and Anti-dsDNA(+) Women −86 ANA(+) and Anti-dsDNA(−) Women	-121 ANA(−) and Anti-dsDNA(−) Women	11.5% 30.2%	47.10%	0% 18.6%	36.30%	100% 38.5%	22.80%
Li et al., 2015 [77]	Not provided	−ANA	−204 ANA(+) Women	-313 ANA(+) Women	27.72%	45.03%	21.78%	43.50%	21.43%	3.39%
Ying et al., 2013 [79]	Retrospective	−ANA −ACA	−20 ANA(+) and ACA(+) Women −51 ANA(+) and ACA(-) Women	−116 ANA(−) and ACA(−) Women	25% 35.3%	53.40	n/a	n/a	n/a	n/a
Zhu et al., 2013 [80]	Retrospective	−ANA	−66 ANA(+) Women	−233 ANA(−) Women	17.30%	56.50%	9.61%	51.10%	44.40%	9.62%
Ying et al., 2013 [78]	Not provided	−ANA	−50 ANA(+) Women	−50 ANA (−) Women	28%	52%	n/a	n/a	n/a	n/a
Ying et al., 2012 [68]	Not provided	−ANA	−66 ANA(+) Women	−233 ANA(−) Women	28.10%	46.40%	n/a	n/a	n/a ^a^	n/a ^a^

^a^: In Ying et al., 2012 study miscarriage rates was provided per gestational sacks, thus, information on miscarriages per clinical pregnancy could not be extracted.

**Table 4:** Study characteristics regarding auto-antibodies affecting the reproductive system.

Studies	Type of Study	Type of Auto-Antibodies	Study Group	Control Group	Clinical Pregnancy Rate		Live Birth/Ongoing Pregnancy Rate		Miscarriage Rate	
					Study Group	Control Group	Study Group	Control Group	Study Group	Control Group
Rogenhofer et al., 2015 [82]	Case Report	−AGA	n/a	n/a	n/a	n/a	n/a	n/a	n/a	n/a
Subit et al., 2011 [83]	Prospective	−AEA	−AEA+ 296 Single IUI cycles 105 double IUI cycles	AEA− 673 single IUI cycles 169 double IUI cycles	4.01% 14.29%	13.07% 16.57%	n/a	n/a	n/a	n/a
Zini et al., 2011 [91]	Prospective	−ASA	−26 ASA(+) Men	−225 ASA(−) Men	42%	52%	n/a	n/a	n/a	n/a
Caccavo et al., 2011 [85]	Prospective	aLN-1	−11 aLN-1 positive women with endometriosis	24 aLN-1 negative women with endometriosis	n/a	n/a	36%	29%	n/a	n/a
Sarapik et al., 2010 [86]	Not provided	−AEA	−190 AEA positive women	n/a	OR: IgA: 0.95 IgG: 1.00 ^a^	n/a	n/a	n/a	n/a	n/a
Francavilla et al., 2009 [87]	Cross-Over	−ASA	−38 ASA positive men	−212 ASA negative OAT men	19% (without COH) 17.6% (with COH)	2.9% (without COH) 6.4% (with COH	n/a	n/a	n/a	n/a
Randall et al., 2009 [88]	Not provided	AEA	Not Provided ^b^							
Van Weert et al., 2008 [89]	Prospective	ASA	−43 ASA positive men	−430 ASA negative men	20.93%	25.76%	n/a	n/a	n/a	n/a
Esteves et al., 2007 [90]	Retrospective	−ASA	17 men with 50% or more ASA	301 men with 20% or less ASA	47.06%	53.16%	n/a	n/a	25%	17.94%

^a^: The study provided only odds ratio for IgA and IgG, as well as certain molecular weight IgAs and IgGs; ^b^:The raw data of the study could not be obtained. A total of 352 patients were included and miscarriage was higher in the AEA positive group.

## Supplementary Materials

Supplementary materials can be found at http://www.mdpi.com/1422-0067/20/4/892/s1.
