# Supplementary material for: The Impact of Autoantibodies on IVF Treatment and Outcome: A Systematic Review
Source: Int J Mol Sci. 2019 Feb 19;20(4):892. doi: 10.3390/ijms20040892 (PMC6412530; doi:10.3390/ijms20040892)
Supplement: Supplementary file 1 [file ijms-20-00892-s001.pdf]

Supplemetary: Search Strategy employed

1. In Vitro Fertilization
2. Intracytoplasmic Sperm Injection
3. Intrauterine insemination
4. IVF
5. ICSI
6. IUI
7. Assisted Reproduction
8. #1 OR #2 OR #3 OR #4 OR #5 OR #6 OR #7
9. Immune system
10. Immune disorders
11. Antibodies
12. Autoantibodies
13. Autoimmune disorders
14. Anti-nuclear antibodies
15. Antisperm antibodies
16. Antithyroid antibodies
17. Antiphospholipid antibodies
18. #9 OR #10 OR #11 OR #12 OR #13 OR #14 OR #15 OR #16 OR #17
19. #8 AND #18
